# Anterior-posterior view by full-body digital X-ray to rule out severe spinal injuries in Polytraumatized patients

**DOI:** 10.1186/s12873-021-00419-1

**Published:** 2021-03-05

**Authors:** Sonja Häckel, Elena Hofmann, Helen Anwander, Christoph E. Albers, Jasmin Basedow, Sebastian F. Bigdon, Aristomenis K. Exadaktylos, Marius J. B. Keel, Robert N. Dunn, Sithombo Maqungo, Lorin M. Benneker, Michael Held, Sven Hoppe

**Affiliations:** 1 Department of Orthopaedic Surgery and Traumatology, Inselspital, Bern University Hospital, University of Bern, Freiburgstrasse 18, 3010 Bern, Switzerland; 2 Department of Radiology, Sonnenhof Hospital, Buchserstrasse 30, 3006 Bern, Switzerland; 3 Emergency Department, Inselspital, Bern University Hospital, University of Bern, Freiburgstrasse 18, 3010 Bern, Switzerland; 4 Trauma Center Hirslanden, Clinik Hirslanden Zurich, Witellikerstrasse 40, 8032 Zurich, Switzerland; 5 Orthopaedic Research Unit, Department of Orthopaedic Surgery, Groote Schuur Hospital and Red Cross Children’s Hospital, University of Cape Town, Klipfontein Rd, Rondebosch, Cape Town, 7700 South Africa

**Keywords:** Spinal injuries, LODOX-Statscan, Diagnostic accuracy, Full-body digital X-ray, Radiography

## Abstract

**Background:**

Spinal injuries are present in 16–31% of polytraumatized patients. Rapid identification of spinal injuries requiring immobilization or operative treatment is essential. The Lodox-Statscan (LS) has evolved into a promising time-saving diagnostic tool to diagnose life-threatening injuries with an anterior-posterior (AP)-full-body digital X-ray.

**Methods:**

We aimed to analyze the diagnostic accuracy and the interrater reliability of AP-LS to detect spinal injuries in polytraumatized patients. Therefore, within 3 years, AP-LS of polytraumatized patients (ISS ≥ 16) were retrospectively analyzed by three independent observers. The sensitivity and specificity of correct diagnosis with AP-LS compared to CT scan were calculated. The diagnostic accuracy was evaluated by using the area under the ROC (receiver operating characteristic curve) for sensitivity and specificity. Interrater reliability between the three observers was calculated using Fleiss’ Kappa. The sensitivity of AP-LS was further analyzed by the severity of spinal injuries.

**Results:**

The study group included 320 patients (48.5 years ±19.5, 89 women). On CT scan, 207 patients presented with a spinal injury (65%, total of 332 injuries). AP-LS had a low sensitivity of 9% (31 of 332, range 0–24%) and high specificity of 99% (range 98–100%). The sensitivity was highest for thoracic spinal injuries (14%). The interrater reliability was slight (κ = 0.02; 95% CI: 0.00, 0.03). Potentially unstable spinal injuries were more likely to be detected than stable injuries (sensitivity 18 and 6%, respectively).

**Conclusion:**

This study demonstrated high specificity with low sensitivity of AP-LS in detecting spinal injuries compared to CT scan. In polytraumatized patients, AP-LS, implemented in the Advanced Trauma Life Support-algorithm, is a helpful tool to diagnose life-threatening injuries. However, if spinal injuries are suspected, performing a full-body CT scan is necessary for correct diagnosis.

**Supplementary Information:**

The online version contains supplementary material available at 10.1186/s12873-021-00419-1.

## Key points

- The average sensitivity of anterior-posterior Lodox-Statscan for spinal injuries was 9% (1–14% depending on the spinal region) with a high overall specificity > 98%
- The overall interrater reliability was slight (slight κ = 0.02); the radiology attending showed the highest sensitivity for detecting spinal injuries.
- The sensitivity of anterior-posterior Lodox-Statscan was higher for potentially unstable injuries (18%) compared to stable injuries (6%)

## Introduction

Spinal injuries are a common finding in polytraumatized patients with an incidence of 18–40% [1, 2]. The early identification of spinal injuries is critical in the initial management of the trauma patient to avoid adverse events due to incorrect immobilization and mismanagement [3, 4]. According to the guidelines of the National Institute for Clinical Excellence (NICE), spinal injuries are suspected if a patient has any significant distracting injuries, a reduced level of consciousness or is under the influence of drugs or alcohol, which might be associated with confusion or uncooperativeness. Moreover, in the clinical examination, a spinal injury is assumed when a patient suffers from any spinal pain, hand or foot weakness or altered sensation, priapism (unconscious or exposed male) or a history of past spinal problems, including previous spinal surgery or conditions that predispose to the instability of the spine [5]. If any of these criteria are met, the next step is radiological imaging, which is pivotal for the correct diagnosis and treatment of spinal injuries.

In the quest for improved imaging techniques in the emergency room, the Lodox-Statscan (LS), initially used in the South African mining industry to reduce diamond theft, has evolved into a promising time-saving diagnostic tool [6]. The LS uses a linear scanning technique with a highly collimated (laser-like) X-ray fan beam, which spreads out in only one direction. On the contrary, conventional X-ray systems use a wide cone-beam around the primary photons, which causes more room scatters and increases overall patient radiation. The translating C-arm of the LS allows imaging angles between 0° (AP view) and 90° (lateral view). The X-ray tube, X-ray fan beam, collimating slit and detector all move together along a linear scanning path, producing images from 100 mm/ 4in square and up to 1800 mm/70 in by 680 mm/ 27 in compared to approximately 400 mm/ 16 in square by conventional X-ray systems [7]. An anterior-posterior (AP) full-body scan by LS with minimal radiation dose is completed within 13 s [6], imaging in two planes within 3 to 5 minutes [8].

Especially in a setting challenged by high patient numbers and limited physical and human resources, the high speed of imaging allows a reduction in resuscitation time [9]. In 2007, routine LS was implemented in the modified Bernese Advanced Trauma Life Support (ATLS), replacing the conventional radiographs of the lateral cervical spine, AP-chest, and AP -pelvis [10]. A second plane is usually performed if there are concerns for an immediate CT scan such as a pregnancy or a highly unstable patient. While many studies have compared CT scans and conventional radiographs as a diagnostic tool [11–13], only a limited number of studies validated the diagnostic accuracy of the LS. These studies have reported a sensitivity of 49–83% and specificity of 95–100% for the diagnosis of spinal injuries on LS [14, 15]. However, in our study, we included a higher number of patients and also analyzed the interrater reliability. To our knowledge, no study has analyzed the diagnostic accuracy of AP-LS for spinopelvic injuries.

The objective of the current study was to evaluate the AP-LS as a diagnostic tool for spinal injuries in a Level 1 trauma center using a CT scan as the reference method. Therefore, we analyzed sensitivity and specificity and interrater reliability of AP-LS to detect spinal injuries specifically for cervical, thoracolumbar and spinopelvic injuries.

## Materials and methods

All methods were carried out in accordance with relevant guidelines and regulations. General consent of patients was obtained. The institutional review board (Health and Welfare Directorate of the Canton of Bern, Switzerland; Cantonal Ethics committee for research, Project ID 2019–02142) waived the need for informed consent. All methods were carried out in accordance with relevant guidelines and regulations. All the experimental protocols were approved by the institutional review Board (Health and Welfare Directorate of the Canton of Bern, Switzerland; Cantonal Ethics committee for research, Project ID 2019–02142).

### Patients

The study group was a consecutive series of polytraumatized patients admitted to our Level I trauma center. The inclusion criterion was solely an Injury Severity Score (ISS) ≥16.

Between 02/2009 and 12/2012, 344 patients aged 16 years and older with an ISS equal to or greater than 16 underwent AP-LS and a full-body CT scan upon presentation in the emergency department. Data were retrieved from individual patient records and the picture archiving and communication system (PACS) image software (Sectra Workstation IDS7, Version 19.3, Sectra AB© Sweden). Independent variables included age, sex, mechanism of injury, and ISS.

### Sample size calculation

The study will focus on a total of 335 participants. This is a diagnostic test accuracy study to estimate the sensitivity and specificity of LS in detecting fracture. Diagnostic measures will be estimated with 95% Wilson confidence intervals. We expect the prevalence of fracture to be between 20 and 40% [1, 16], sensitivity to be between 40 and 70% [1] and specificity between 85 and 100% [1]. A sample of 335 participants will result in a two-sided 95% Wilson confidence interval around the sensitivity and specificity as shown in the tables below (Supplemental material Table 1a Sensitivity table; Table 1b Specificity table).

### Radiographic imaging

The AP-LS was performed by an LS (Statscan Critical Imaging System, Lodox Systems [Pty] Ltd., Johannesburg, South Africa). The LS C-arm rotates around the patient with an angle between 0 and 90 degrees and can provide an AP view within 13 s (138 mm/s) [1]. The patient was positioned with the upper extremities lateral to the body to avoid an overlay with the thorax, spine and pelvis. Detailed information about the LS Linear Slot Scanning Radiography System can be found at the companies online presence (http://lodox.com).

A full-body CT scan followed the performance of an LS. All CT examinations were performed using a 16-slice multidetector-row computed tomography system (Sensation 16, Siemens, Forchheim, Germany) with collimation of 16 by 1.5 mm and a reconstruction slice thickness of both 2 mm and 5 mm.

The full-body CT scans of all patients were analyzed in terms of spinal injuries by two independent (blinded) investigators. All injuries were classified according to the AO-Classification for spinal injuries [17–19]. If the classification was not concordant, the two investigators reached a consensus. The results of the CT scans were used as the diagnostic reference.

### Image analysis

The full-body CT scans of all patients were analyzed in terms of spinal injuries by two independent (blinded) investigators. These were both senior physicians: An experienced radiologist and an experienced spine surgeon. All investigators went over various planes (ap, lateral and sagittal planes) while evaluating the CT scans for spinal injuries.

The AP-LS was assessed for signs of spinal injuries by three independent observers (physicians with the following speciality and experience: radiology attending with > 5 years of experience, orthopedic attending with > 5 years of experience, and orthopedic resident with < 5 years of experience). If any spinal injuries were visible on AP-LS, the number (some patients presented with multiple spinal injuries) and level(s) of the spinal injury were noted. The level of the injury was classified as cervical, thoracic, lumbar, or sacropelvic.

### Data sharing statement

All data generated or analyzed during the study are included in the published paper.

### Statistical analysis

SPSS (IBM SPSS Statistics for Windows, Version 25.0, IBM Corp, Armonk, NY, 2017) was used to perform the statistical analyses.

Diagnostic accuracy (sensitivity, specificity, negative, and positive predictive values) was calculated for each of the following levels: cervical (occipitocervical and subaxial combined), thoracic, lumbar spine, and sacropelvic. The AUC (Area under the operator receiver characteristics curve) was computed for the three observers, namely the radiology attending (RA), orthopedic resident (OR), and the orthopedic attending (OA). The AUC ranges from 0.5–1. Values of 0.9–1.0 show that the test has an excellent discrimination ability, whereas values of 0.8–0.9 demonstrate a good, 0.7–0.8 a fair, 0.6–0.7 a poor, and 0.5–0.6 fail discrimination ability of the test [20].

The sensitivity and specificity were further calculated for stable versus potentially unstable injuries. Stable injuries were defined as the following: A0-, A1- and A2- type subaxial and thoracolumbar injuries and: A- and B-type sacropelvic injuries. Potentially unstable injuries were defined as the following: A3 and A4-type as well as B- and C-type subaxial and thoracolumbar injuries, and C-type sacropelvic injuries according to AO-Spine classification [21]. If a patient presented with a stable and potentially unstable fracture, he or she was allocated as potentially unstable.

Interrater reliability between the three observers was calculated using Fleiss’ Kappa and rated, according to Landis and Koch [22]. Kappa values range from − 1 to + 1 and are interpreted as follows: < 0.00 poor, 0.00–0.20 slight, 0.21–0.40 fair, 0.41–0.60 moderate, 0.61–0.80 substantial, and 0.81–1.00 almost perfect interrater reliability.

## Results

### Patients demographics

**Fig. 1:**
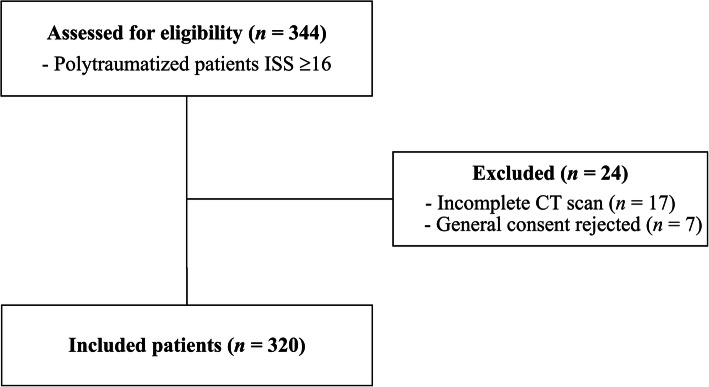
Patient selection flowchart

Three hundred forty-four consecutive patients were eligible for inclusion. Twenty-four patients were excluded due to incomplete CT scan (*n* = 17) or missing informed consent (*n* = 7). Three hundred twenty patients (332 spinal injuries; mean age: 48 ± 19 years; range 17–89 years; 89 women) were included. An overview of the case selection process (Fig. 1) and patient demographics are shown (Table 1).

**Table 1:** Patients’ Characteristic**s**

Characteristic	Total (*n* = 320)
Patients’ characteristics	
Age (y)^a^	48 ± 19
Sex	
Women	89
Men	231
ISS^b^	22 (± 8)
Spinal Injury	
Yes	207 (65)
No	113 (35)
Injury mechanism	
Fall from height	104 (33)
Traffic accidents by	
- Car	53 (17)
- Bike	34 (11)
- Motor vehicle	27 (8)
- Pedestrian	22 (7)
Ski accident	12 (4)
Paragliding accident	12 (4)
Other	56 (18)
Total spinal injuries	*n* = 332
Cervical	75 (23)
Thoracic	97 (29)
Lumbar	94 (28)
Sacropelvic	66 (20)

^a^Data are mean ± standard deviation with percentages in parentheses. ^b^Data are median with interquartile range in parentheses. *ISS* Injury Severity Score

### The diagnostic accuracy of the anterior-posterior full-body digital X-ray (Lodox Statscan) for the different levels of spinal injuries

The overall sensitivity of AP-LS was 9%, with an overall specificity of 99%. Depending on the injury level, sensitivity was between 1 and 14%, specificity between 98 and 100% (Table 2). The sensitivity was lowest for cervical spinal injuries (0–3%) and highest for thoracic spinal injuries (2–20%). The overall positive predictive value (PPV) was 63%, and the negative predictive value (NPV) was 76%. Depending on the level of injury, PPV and NPV ranged between 58 and 67% and 72–80%, respectively (Table 2).

**Table 2:** Diagnostic accuracy of the Lodox Statscan in the detection of cervical (occipitocervical and subaxial), thoracic, lumbar, and sacropelvic injuries

Region and Observer	Sensitivity (%)	Specificity (%)	PPV (%)	NPV (%)	AUC
**Cervical Spine**					
Radiology Attending	1 (1/75) [0–8]	100 (245/245) [98–100]	100 (1/1) [5–100]	77 (245/319) [0–95]	0.51 [0.43–0.58]
Orthopaedic Resident	0 (0/75) [0–6]	99 (243/245) [97–100]	0 (0/2) [0–80]	76 (243/318) [71–81]	0.50 [0.42–0.57]
Orthopaedic Attending	3 (2/75) [0–10]	100 (245/245) [98–100]	100 (2/2) [20–100]	77 (245/318) [0–80]	0.51 [0.44–0.59]
**Thoracic Spine**					
Radiology Attending	19 (18/97) [12–28]	99 (221/223) [96–100]	90 (18/20) [67–98]	74 (221/300) [68–78]	0.59 [0.52–0.66]
Orthopedic Resident	2 (2/97) [0–8]	99 (220/223) [96–100]	40 (2/5) [7–83]	70 (220/315) [64–75]	0.50 [0.43–0.57]
Orthopedic Attending	20 (19/97) [12–29]	96 (213/223) [92–98]	66 (19/29) [46–81]	73 (213/291) [68–78]	0.57 [0.50–0.65]
**Lumbar Spine**					
Radiology Attending	18 (17/94) [11–28]	99 (224/226) [96–100]	90 (17/19) [65–98]	74 (224/301) [69–79]	0.59 [0.51–0.66]
Orthopedic Resident	1 (1/94) [0–6]	97 (219/226) [93–99]	13 (1/8) [0–53]	70 (219/312) [65–75]	0.49 [0.42–0.56]
Orthopedic Attending	18 (17/94) [11–28]	97 (219/226) [93–99]	71 (17/24) [49–87]	74 (219/296) [69–79]	0.58 [0.50–0.65]
**Sacrum**					
Radiology Attending	24 (16/66) [15–37]	97 (242/250) [94–99]	67 (16/24) [45–84]	82 (242/296) [78–87]	0.61 [0.52–0.69]
Orthopedic Resident	2 (1/66) [0–9]	100 (249/250) [97–100]	50 (1/2) [3–97]	78 (249/318) [74–84]	0.51 [0.43–0.58]
Orthopaedic Attending	3 (2/66) [0–11]	100 (249/250) [97–100]	67 (2/3) [13–98]	79 (249/317) [75–84]	0.51 [0.43–0.59]

Data in parentheses are numerators and denominators with 95% confidence intervals in brackets. PPV Positive predictive value, NPV *Negative predictive value,* AUC Area under the operator receiver characteristics curve

The overall AUC was < 0.7 (0.49–0.61), independent of the injury level, demonstrating a poor value of the AP-LS as a diagnostic instrument in the case of suspected spinal injuries (Table 2).

**Fig. 2:**
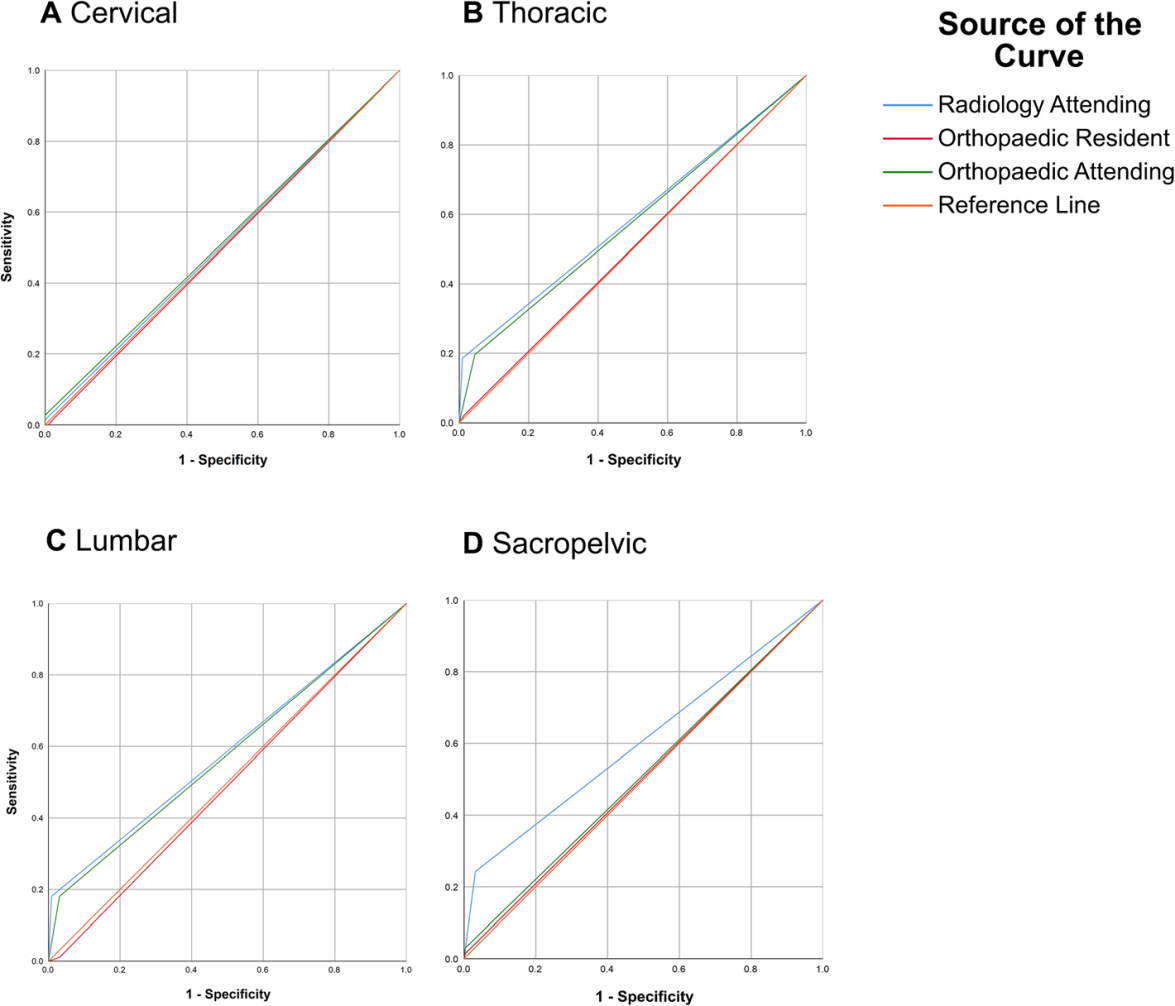
Receiver operating characteristic (ROC) curves for the diagnostic accuracy of the Lodox Statscan (LS) in the detection of spinal injuries depending on the injury level. ROC graphs illustrate the relative values of specificity and sensitivity for all observers

We summarized occipitocervical and subaxial injuries to cervical spinal injuries because the three clinical observers were asked to rate cervical spine injuries. The AUC was lowest for cervical spinal injuries (0.51 [95% CI: 0.43,0.58]) and highest for thoracic (0.55 [95% CI: 0.48,0.63] and lumbar spinal injuries (0.55 [95% CI. 0.48,0.63]. The RA presented the highest values of AUC (0.58 [95% CI: 0.50,0.65], whereas the OR attained the lowest values (0.50 [95% CI: 0.43,0.57] (Table 2, Fig. 2).

### The sensitivity of the anterior-posterior full-body digital X-ray (Lodox Statscan) for stable and potentially unstable injuries of the spine

The sensitivity of potentially unstable injuries was higher compared to stable injuries. The difference was highest in potentially unstable sacropelvic injuries with a 5% (95% CI: 2,17) sensitivity in stable injuries versus 26% (95% CI: 13,45) for potentially unstable injuries. Similar trends were observed in the thoracic and lumbar spinal regions: 6% (95% CI:13,35) in stable thoracic versus 21% (95% CI:2,19) in potentially unstable thoracic injuries, and 20% (95% CI:5,20) in potentially unstable versus 10% (95% CI:5,20) in stable lumbar spinal injuries. Since it is difficult to clearly assign occipitocervical injuries to stable or unstable, only the overall sensitivity was given for these types of injuries. Only one C1 ring injury of the upper cervical spine was correctly identified, which leads to low sensitivity of 1% (95% CI:0,13). In subaxial spinal injuries, only a slight difference of 1% (95% CI:0,16] in stable versus 4% (95% CI:0,30) in potentially unstable spinal injuries was evident (Table 3).

**Table 3:** Sensitivity of AP-LS for the detection of spinal injuries. Injuries were classified as stable (subaxial and thoracolumbar: A0-, A1- and A2- type injuries; sacropelvic: A- and B-type injuries) and potentially unstable spinal injuries (subaxial and thoracolumbar: A3 and A4-type injuries, B- and C-type injuries; sacropelvic: C-type injuries) according to AO-Spine classification [21]. Since it is difficult to clearly assign occipitocervical injuries to stable or unstable, only the overall sensitivity was given for these types of injuries

Region and Observer	Sensitivity (%)
**Occipitocervical**	
*Stable and Potentially Unstable Injuries*	
Radiology Attending	0 (0/41) [0–9]
Orthopedic Resident	0 (0/41) [0–9]
Orthopedic Attending	2 (1/41) [0–13]
**Subaxial Spine**	
*Stable Injuries*	
Radiology Attending	0 (0/31) [0–14]
Orthopedic Resident	0 (0/31) [0–14]
Orthopedic Attending	3 (1/31) [0–19]
*Potentially Unstable Injuries*	
Radiology Attending	6 (1/17) [0–31]
Orthopedic Resident	0 (0/17) [0–29]
Orthopedic Attending	6 (1/17) [0–31]
**Thoracic Spine**	
*Stable Injuries*	
Radiology Attending	6 (3/47) [2–19]
Orthopedic Resident	4 (2/47) [1–16]
Orthopedic Attending	6 (3/47) [2–19]
*Potentially Unstable Injuries*	
Radiology Attending	31 (15/48) [19–46]
Orthopedic Resident	0 (0/48) [0–9]
Orthopedic Attending	33 (16/48) [21–49]
**Lumbar Spine**	
*Stable Injuries*	
Radiology Attending	17 (12/72) [9–28]
Orthopedic Resident	1 (1/72) [0–9]
Orthopedic Attending	13 (9/72) [6–23]
*Potentially Unstable Injuries*	
Radiology Attending	23 (5/22) [9–46]
Orthopedic Resident	0 (0/22) [0–18]
Orthopedic Attending	36 (8/22) [18–59]
**Sacrum**	
*Stable Injuries*	
Radiology Attending	14 (7/53) [6–26]
Orthopedic Resident	1 (1/53) [0–11]
Orthopedic Attending	1 (2/53) [0–14]
*Potentially Unstable Injuries*	
Radiology Attending	78 (9/13) [39–90]
Orthopedic Resident	0 (0/13) [0–28]
Orthopedic Attending	0 (0/13) [0–28]

Data in parentheses are numerators and denominators with 95% confidence intervals in brackets

### Interrater reliability

Interrater reliability was calculated for each level of spinal injuries. Results revealed poor interrater reliability for cervical spinal injuries (κ = − 0.01; 95% CI: − 0.07, 0.06), the interrater reliability was fair for thoracic (κ = 0.22; 95% CI: 0.15, 0.28) and slight for lumbar (κ = 0.19; 95% CI: 0.13, 0.26) and sacropelvic spinal injuries (κ = 0.04; 95% CI: − 0.02, 0.10). The mean interrater reliability, independent of the spine region, was slight (κ = 0.02; 95% CI: 0.00, 0.03).

**Fig. 3:**
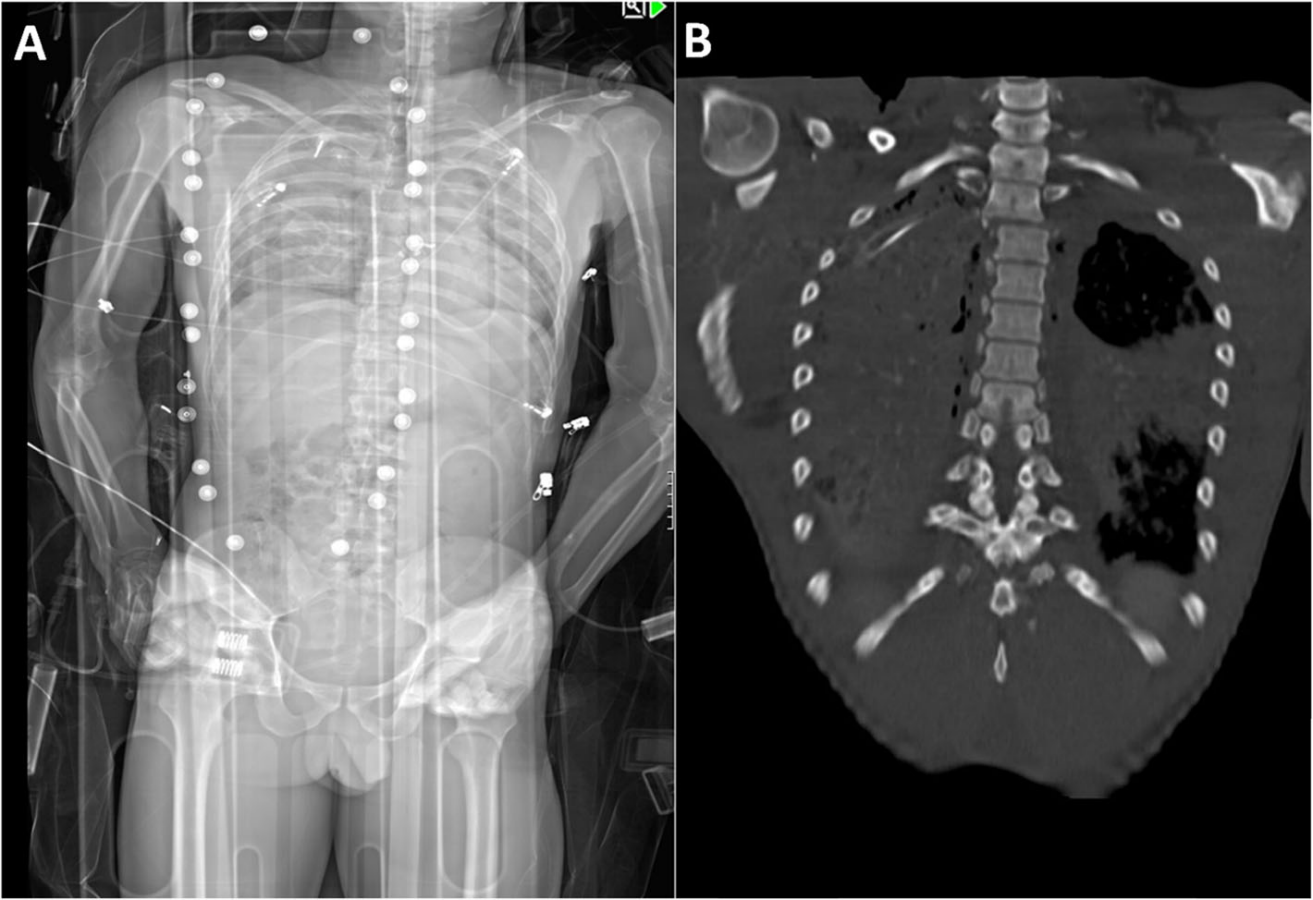
Example of a missed unstable injury using anterior-posterior Lodox Statscan (AP-LS). An unstable spinal injury fo the second and third thoracic vertebra shown in AP-LS (**a**), and the full-body CT scan (**b**) of 46-year-old men after falling from a height. None of the three observers identified the injury in AP-LS

**Fig. 4:**
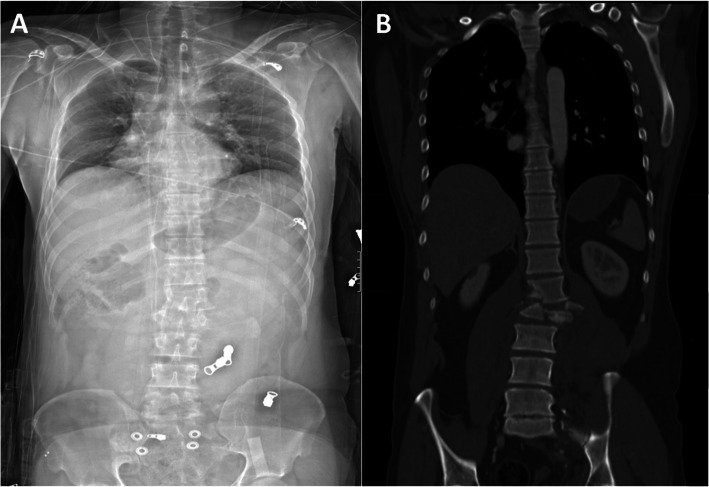
Example of a correctly identified unstable injury using anterior-posterior Lodox Statscan (AP-LS). C-type injury of the 12th thoracic and first lumbar vertebra shown in AP-LS (**a**) and the corresponding full-body CT scan (**b**) of 36-year-old men after a car accident. This injury was correctly identified by two of the three observers (Radiologist and Orthopedic attending)

Examples of a misdiagnosed and correctly identified injury are shown in Figs. 3 and 4.

## Discussion

In the present study, 64% (207/320) of polytraumatized patients (ISS ≥ 16) presented with spinal injuries, diagnosed by CT scan. Our results showed a low diagnostic accuracy of the sole anterior-posterior LS in the detection of spinal injuries. The overall sensitivity of the anterior-posterior LS was low (1–14%) with high specificity (98–100%) independent of the injury level. The positive and negative predictive values were 58–67% and 72–80%, respectively. ROC-curves and interrater reliability (of three blinded observers) displayed a low diagnostic value of anterior-posterior LS in the case of suspected spinal injuries.

One of the biggest advantages of the LS is the time and dose savings compared to a CT scan and conventional radiography. For the LS, there is neither a need to undress nor to transfer the patient to another table. With the C-Arm running around the patient, an AP view of the whole body takes less than 13 s and an additional maximum of 15 s to create an image on the screen [7]. Especially in an emergency setting, there is an urgency of completing the full (radiological) diagnostic. It has been shown that the delay of the full-body CT scan after conventional x-rays of the chest and pelvis (as recommended in the ATLS-algorithm) is about 47 min [23]. This delay can be decreased by performing AP-LS imaging (the time span between AP-LS and a full-body CT scan is about 37 min) [15]. Besides, LS has a low dose of radiation because of the collimated x-ray fan beam. The radiation dose for an AP chest and AP pelvis view is 680 μSv and hence, much higher when compared to an AP-LS with 99.15 μSv [24]. Therefore LS dose is (as a percentage of conventional x-ray dose) 72% (chest) and 2% (pelvis) [25].

To our knowledge, this is the first study, including over 300 polytraumatized patients with three blinded, independent examiners who assessed AP-LS for spinal injuries. Other studies assessing the accuracy of (AP-)LS compared to CT scans included between 184 and 245 polytraumatized patients [1, 15, 26]. All studies compared LS with CT scanning, to determine the sensitivity [1] and diagnostic accuracy [15, 26] of LS investigation in detecting injuries to the chest, thoracolumbar spine, and pelvis. In addition, a recent study by Yang et al. evaluated the available evidence for the clinical effectiveness and biohazard safety of the LS in acute medical emergencies [14]. The assessments of the included studies were mainly done by AP-LS, out of these studies only one stated, that in total 8 patients received an additional lateral view. In comparison to these studies, our results revealed a lower overall sensitivity of AP-LS of 9% compared to 70% [14, 26], 59% [1, 14], and 49% [15]. However, a similarly high specificity, compared to our results, of 98–100% was found [14, 15]. Additionally, the same tendencies with the lowest sensitivity for cervical (1% compared to 57% [26]) and higher for thoracolumbar spinal injuries (13% compared to 74–83% [1, 26]) were reported. We did not find previous results for sacropelvic injuries. This wide range of diagnostic accuracy was addressed before. In the review of Yang et al. [14], they stated on a notable risk of bias of the individual studies evaluating the LS diagnostic capability.

However, two other studies have examined the accuracy of injury detection (not spine specific) by clinicians using AP-LS, reporting an overall fracture-site dependent sensitivity of 89% [15, 27]. However, these studies did not assess the interrater reliability [15] or did not have a CT scan of all patients [27]. In our study, we choose two orthopedic surgeons and one radiologist for the evaluation of spinal injuries. In our institution, it is standard that a radiologist prepares a written report and an orthopedic assistant doctor (resident), together with an experienced orthopedic spinal surgeon, evaluates all images as a basis for the indication for treatment. The experienced attendings from both radiology and orthopedics showed a higher sensitivity compared to the orthopedic resident. Our findings are consistent with the results of Holdt et al. [27]. They showed for peripheral skeletal injuries that the diagnostic accuracy of LS highly depends on the expertise of the evaluating clinician as well as the clinical suspicion and trauma mechanism [27].

The current study demonstrated higher sensitivity for potentially unstable compared to stable spinal injuries, with the identification of potentially unstable spinal injuries being of high clinical relevance. Nevertheless, a fair amount of potentially unstable injuries of the thoracolumbar spine could not be detected by the AP-LS (Fig. 3). Similar results were reported by Deyle et al. with a distinctive proportion of unstable thoracic spine injuries (76%) that required stabilization [1]. Moreover, the diagnostic accuracy was lowest for the cervical spine, including the occipitocervical spine (C0/C1 and C1/C2). The sub-analyses showed that only one of 41 injuries of the upper cervical spine was correctly identified. Even in non-polytraumatized patients, cervical spine injuries can be difficult to diagnose on plain radiographs [28]. Often, special x-rays, especially for the occipitocervical spinal region (e.g. transoral), are needed to rule out bony injuries or injuries to the atlanto-dental and -occipital ligaments. The majority of injuries to the cervical spine are discogenic and/or ligamentous injuries, which can usually need loaded (flexion/extension) x-rays to be diagnosed. Overall, another possible explanation could be the higher overlying of bones and organs in combination with artifacts from, e.g., a stiff neck or clothes, especially at the cervical spine (Fig. 3).

The main strength of our study was the high number of polytraumatized patients with suspected spinal injuries. Another strength was the number of observers, who are of different specialties and training levels (radiology attending, orthopedic attending and orthopedic resident), as well as blind to the clinical information. In the study of Deyle et al., the preliminary diagnosis was made by a physician, followed by a definitive diagnosis by a radiologist [1]. In the study by Jöres et al., all AP-LS were examined by two radiologists [15], whereas Chen et al. did not describe the number and specialty of the observer. One other study with two musculoskeletal radiologists focused on the diagnostic of the pelvis and appendicular skeleton, but not on spinal injuries [29]. Besides, we provided the first data on the diagnostic accuracy of AP-LS on sacropelvic injuries.

The main limitation of our study is the missing lateral LS plane. The sensitivity for the detection of spinal injuries might be higher, especially for A- or B-type injuries. Because lateral planes are not included in our standard clinical protocol, this plane is missing in most patients. Therefore, we could not implement this data into our analysis. Other limitations of the study are, that we did not evaluate the patients’ clinical outcomes as well as the cost-effectiveness.

## Conclusion

This study demonstrated a high specificity but low overall sensitivity and diagnostic accuracy of the sole anterior-posterior LS in the detection of cervical, thoracic, lumbar, or sacropelvic spinal injuries. In the polytraumatized patient, anterior-posterior LS imaging within the Advanced Trauma Life Support-algorithm is a helpful tool to diagnose life-threatening injuries, especially in the detection of chest and extremity injuries and in a setting challenged by high patient numbers [10]. However, if spinal injuries are suspected, performing a full-body CT scan is mandatory for a correct diagnosis.

## Supplementary Information

**Additional file 1.** Sample size calculation.

## Data Availability

The datasets used and/or analysed during the current study are available from the corresponding author on reasonable request.

## Abbreviations

AP-LS: Anterior-posterior Lodox-Statscan
AUC: Area under the operator receiver characteristics curve
CI: Confidence interval
ATLS: Advanced Trauma Life Support
ISS: Injury Severity Score
LS: Lodox-Statscan
OA: Orthopedic attending
OR: Orthopedic resident
RA: Radiology attending
ROC: Receiver operating characteristic

## Declarations

All methods were performed in accordance with relevant guidelines and regulations.
