# Supplementary material for: Anterior-posterior view by full-body digital X-ray to rule out severe spinal injuries in Polytraumatized patients
Source: BMC Emerg Med. 2021 Mar 5;21:27. doi: 10.1186/s12873-021-00419-1 (PMC7934441; doi:10.1186/s12873-021-00419-1)
Supplement: Supplementary file 1 — Additional file 1. Sample size calculation. [file 12873_2021_419_MOESM1_ESM.docx]

**Anterior-Posterior View by Full-Body Digital X-Ray to Rule Out Severe Spinal Injuries in Polytraumatized Patients**

Original Research Article

*Sonja Häckel, MD, [sonja.haeckel@insel.ch](mailto:sonja.haeckel@insel.ch) ^1^

*Elena Hofmann, MD [elena.hofmann@rwth-aachen.de](mailto:elena.hofmann@rwth-aachen.de) ^1^

Helen Anwander, MD [helen.anwander@insel.ch](mailto:helen.anwander@insel.ch) ^1^

Christoph E. Albers, MD [christoph.albers@insel.ch](mailto:christoph.albers@insel.ch) ^1^

Jasmin Basedow, MD, [jasmin.basedow@lindenhofgruppe.ch](mailto:jasmin.basedow@lindenhofgruppe.ch) ^2^

Sebastian F. Bigdon,MD [sebastian.bigdon@insel.ch](mailto:sebastian.bigdon@insel.ch) ^1^

Aristomenis K. Exadaktylos, MD, Prof [aristomenis.exadaktylos@insel.ch](mailto:aristomenis.exadaktylos@insel.ch) ^3^

Marius J. B. Keel, MD, Prof., [mkeel@traumazentrum.ch](mailto:mkeel@traumazentrum.ch) ^4^

Robert N. Dunn, MD, Prof., [robert.dunn@uct.ac.za](mailto:robert.dunn@uct.ac.za) ^5^

Sithombo Maqungo, MD, Prof. [sithombo@msn.com](mailto:sithombo@msn.com) ^5^

Lorin M. Benneker, MD, Prof. [lorin.benneker@insel.ch](mailto:lorin.benneker@insel.ch) ^1^

Michael Held, MD, Prof. [email.held@gmail.com](mailto:email.held@gmail.com) ^5^

Sven Hoppe, MD, [sven.hoppe@insel.ch](mailto:sven.hoppe@insel.ch) ^1^

*Sonja Häckel and Elena Hofmann contributed equally to this work

^1^ Department of Orthopaedic Surgery and Traumatology, Inselspital, Bern University Hospital, University of Bern, Freiburgstrasse 18, 3010 Bern, Switzerland.

^2^ Department of Radiology, Sonnenhof Hospital, Schänzlistrasse 39, 3013 Bern, Switzerland.

^3^ Emergency Department, Inselspital, Bern University Hospital, University of Bern, Freiburgstrasse 18, 3010 Bern, Switzerland.

^4^ Trauma Center Hirslanden, Clinik Hirslanden Zurich, Witellikerstrasse 40, 8032 Zurich, Switzerland.

^5^ Orthopaedic Research Unit, Department of Orthopaedic Surgery, Groote Schuur Hospital and Red Cross Children's Hospital, University of Cape Town, Klipfontein Rd, Rondebosch, Cape Town, 7700, South Africa.

Correspondence to: Sonja Häckel, Department of Orthopaedic and Trauma Surgery, Inselspital, Bern University Hospital, University of Bern, Freiburgstrasse 18, 3010 Bern, Switzerland. Phone +41 31 632 2222; Email to Sonja.haeckel@insel.ch

**Supplemental Material**

**Sample size calculation**

**Table 1a:** Sensitivity table, N=320. 95% Wilson confidence interval (%) varying the prevalence and the expected sensitivity.

|  | | Sensitivity | | | | | | |
| --- | --- | --- | --- | --- | --- | --- | --- | --- |
|  | | 0.4 | 0.45 | 0.5 | 0.55 | 0.6 | 0.65 | 0.7 |
| Prevalence | 0.15 | [27.7; 53.7] | [32.2; 58.5] | [36.8; 63.2] | [41.5; 67.8] | [46.3; 72.3] | [51.3; 76.6] | [56.4; 80.8] |
|  | 0.2 | [29.1; 52] | [33.7; 56.9] | [38.4; 61.6] | [43.1; 66.3] | [48; 70.9] | [53; 75.3] | [58.2; 79.6] |
|  | 0.25 | [30.2; 50.7] | [34.8; 55.6] | [39.5; 60.5] | [44.4; 65.2] | [49.3; 69.8] | [54.3; 74.3] | [59.5; 78.7] |
|  | 0.3 | [31; 49.8] | [35.7; 54.7] | [40.4; 59.6] | [45.3; 64.3] | [50.2; 69] | [55.3; 73.6] | [60.5; 78.1] |
|  | 0.35 | [31.6; 49] | [36.3; 54] | [41.1; 58.9] | [46; 63.7] | [51; 68.4] | [56; 73] | [61.2; 77.5] |
|  | 0.4 | [32.1; 48.5] | [36.8; 53.4] | [41.7; 58.3] | [46.6; 63.2] | [51.5; 67.9] | [56.6; 72.6] | [61.8; 77.1] |

**Table 1b:** Specificity table, N=320. 95% Wilson confidence interval (%) varying the prevalence and the expected specificity.

|  | | Specificity | | | |
| --- | --- | --- | --- | --- | --- |
|  | | 0.85 | 0.9 | 0.95 | 0.99 |
| Prevalence | 0.15 | [80.4; 88.7] | [86; 93] | [91.8; 97] | [97; 99.7] |
|  | 0.2 | [80.2; 88.8] | [85.8; 93] | [91.7; 97] | [96.9; 99.7] |
|  | 0.25 | [80.1; 88.9] | [85.7; 93.1] | [91.6; 97.1] | [96.8; 99.7] |
|  | 0.3 | [79.9; 89] | [85.5; 93.2] | [91.4; 97.1] | [96.7; 99.7] |
|  | 0.35 | [79.7; 89.1] | [85.3; 93.3] | [91.2; 97.2] | [96.6; 99.7] |
|  | 0.4 | [79.4; 89.3] | [85.1; 93.4] | [91.1; 97.3] | [96.4; 99.7] |
